# Uncovering the Genetic Architectures of Quantitative Traits

**DOI:** 10.1016/j.csbj.2015.10.002

**Published:** 2015-11-23

**Authors:** James J. Lee, Shashaank Vattikuti, Carson C. Chow

**Affiliations:** a Department of Psychology, University of Minnesota Twin Cities, Minneapolis, MN 55455, USA; b Mathematical Biology Section, NIDDK/LBM, National Institutes of Health, Bethesda, MD 20892, USA

**Keywords:** Statistical genetics, Quantitative genetics, Population genetics, Average effect of gene substitution, Heritability, GWAS, Compressed sensing, Review

## Abstract

The aim of a genome-wide association study (GWAS) is to identify loci in the human genome affecting a phenotype of interest. This review summarizes some recent work on conceptual and methodological aspects of GWAS. The *average effect of gene substitution* at a given causal site in the genome is the key estimand in GWAS, and we argue for its fundamental importance. Implicit in the definition of average effect is a *linear model* relating genotype to phenotype. The fraction of the phenotypic variance ascribable to polymorphic sites with nonzero average effects in this linear model is called the *heritability*, and we describe methods for estimating this quantity from GWAS data. Finally, we show that the theory of *compressed sensing* can be used to provide a sharp estimate of the sample size required to identify essentially all sites contributing to the heritability of a given phenotype.

## 1 Introduction

The now-classic treatise *Genetics and the analysis of quantitative traits*[1], published three years before the first drafts of the human genome, covered the following sequence of topics:

1. definitions of key quantities in the study of quantitative (continuously varying) traits affected by multiple genetic and environmental causes,
2. methods for estimating some of these quantities without knowledge of the individual genetic sites affecting a given quantitative trait, and
3. the use of DNA-level data to identify the precise genomic regions that contain one or more such polymorphic sites.

In this review we survey work in all of these areas carried out in the decade and a half since the sequencing of the human genome. Modern genotyping technology has enabled genome-wide association studies (GWAS), which have led to a “golden age” of discovery in quantitative genetics [2], and we cannot hope to cover the substantial empirical progress in the identification of genetic loci contributing to quantitative variation. The most that can be done at the outset is to point the reader to the burgeoning research program in which our chosen conceptual and methodological issues are embedded [3], [4], [5], [6], [7], [8], [9], [10].

Much of our discussion can be extended to binary phenotypes (such as disease diagnosis) through the device of treating liability as a quantitative trait affected by multiple genetic and environmental causes.

## 2 The Average Effect of Gene Substitution

We are interested in determining the quantitative influence of a polymorphic site on a given phenotype. Consider a biallelic site with alleles $A_{1}$ and $A_{2}$, where variation potentially affects a phenotype denoted by *Y*. A direct means to determine this quantity is to measure the phenotypic effect of experimentally changing the allelic state of the gene borne by a gamete. Confounding such an experiment, however, is dependence of the phenotypic effect on the allelic states of other genes in the zygote's genome. This nonlinear interaction is called *dominance* if it occurs between genes at the same site but inherited from different parents and *epistasis* if it occurs among genes at different sites. (We follow the classical usage of the term *gene* to refer to a token of heritable material at a given genomic site. Thus, each chromosome contains its own gene.) Fixing the allelic states everywhere else in the genome, we can write the effect of substituting $A_{2}$ for $A_{1}$, as

(1) $$\Delta Y_{A_{1}\to A_{2}|\mathrm{fixed}\;\mathrm{background}}$$

It is not possible to estimate (1) for all backgrounds. There are roughly 10 million single-nucleotide polymorphisms (SNPs) in the human genome where the frequencies of both base pairs (alleles) exceed 0.01. Considering just these polymorphic sites alone, we have a number of multi-SNP genotypes equaling three to the power ten million. The developmental process maps each of these genotypes to an expected phenotypic value, but the astronomically large number of possible genotypes rules out any attempt to estimate this causal mapping in its totality. Even if a given genotype has a relatively high probability, in the sense of containing a common allele at each site, it is quite possible that no individuals in the population actually bear that genotype. Thus, even if it were possible to perform any conceivable mutagenic experiment [11], the sheer number of such experiments would place the genetic architecture of the phenotype—if this is defined by Eq. (1)—hopelessly out of our grasp.

We are thus forced to seek some more tractable object that preserves biological meaning. A natural thought is that we should concentrate on some weighted average of the possible gene substitutions at any given polymorphic site,

(2) $$\alpha =\frac{\sum_{k}w_{k}\Delta Y_{A_{1}\to A_{2}|k}}{\sum_{k}w_{k}}\text{,}$$

where the sums are over all possible configurations (indexed by *k*) of alleles at the other genomic locations. The symbol *α* to represent the *average effect of gene substitution* was first used by Fisher [12]. The weights should take on the same values in the analogous expression defining the gene substitution $A_{2}\to A_{1}$, such that these two quantities have the same absolute value but opposite signs.

**Fig. 1:**
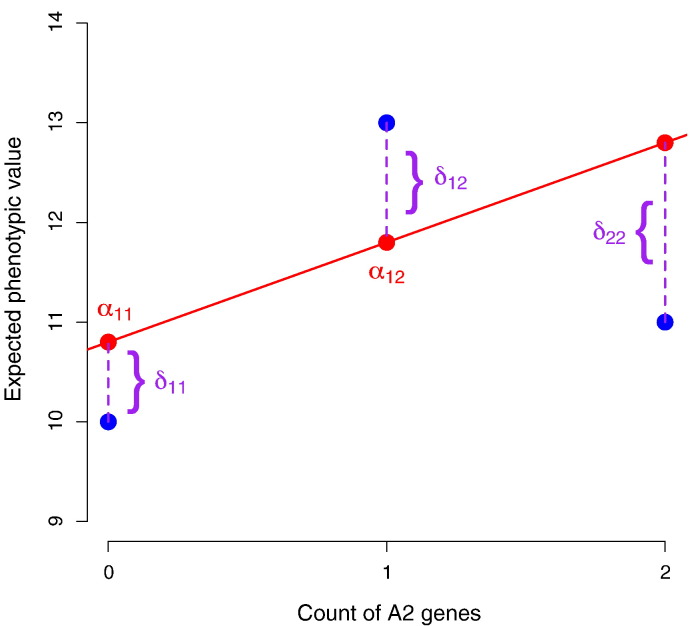
Breeding (additive genetic) values and dominance deviations at a biallelic locus. The frequency of allele $A_{2}$ is 0.6, and the causal effects of $A_{1}A_{1}$ → $A_{1}A_{2}$ and $A_{1}A_{2}$ → $A_{2}A_{2}$ are 3 and − 2 respectively. The genotype frequencies are in Hardy–Weinberg equilibrium. The phenotypic mean of each genotype is equal to the sum of its breeding value (*α*_*ij*_) and genetic residual (*δ*_*ij*_); in this case of nonlinearity within a locus, the genetic residuals are called *dominance deviations*. The phenotypic means are represented by the blue points, and the corresponding breeding values by the red points. The slope of the linear function giving the breeding values is the average effect of gene substitution.

Eq. (2) is an advance only if the weights allow the average to be calculated without knowledge of the myriad addends taking the form of Eq. (1). Fisher defined his average effect of gene substitution such that the weights reproduce the coefficient of the polymorphic site in the multiple regression of the phenotype on all such sites in the genome [13], [14]. To make this equivalence more explicit, let **G** be the vector whose *i*th entry is the expected phenotype obtained by all organisms with a fixed multi-site genotype (arbitrarily labeled as the *i*th) developing within the current range of environmental conditions, **X** the matrix whose *ij*th entry is the number of genes (0, 1, or 2) of the *j*th allelic type present in the *i*th genotype, *α* the vector of average effects, and **R** the vector of residuals (Fig. 1). Without loss of generality, let all variables be standardized. Fisher effectively chose the weights in Eq. (2) such that the sum of the squared residuals,

(3) $${\left\|R\right\|}_{\ell_{2}}^{2}={\left\|G-X\alpha \right\|}_{\ell_{2}}^{2}\text{≡}{\left\|G-A\right\|}_{\ell_{2}}^{2}\text{,}$$

is minimized. Eq. (3) defines a new quantity, *A*_*i*_ = *G*_*i*_ − *R*_*i*_ = ∑_*j*_*X*_*ij*_*α*_*j*_, the *i*th individual's so-called *breeding* or *additive genetic value*. The *ℓ*_2_ norm is the *only* choice of norm in Eq. (3) that leads to the orthogonal decomposition of the total genetic variance,

(4) $$\sigma_{G}^{2}=\sigma_{A}^{2}+\sigma_{R}^{2}\text{.}$$

All other choices will lead to the appearance of the covariance term 2 Cov(*A*, *R*), which essentially implies that the individual's breeding value does not contain all possible information about its phenotypic value that can be obtained from a linear combination of its single-site genotypes; some is abandoned in the residual. Thus, the choice of weights in Eq. (2) following from the use of the *ℓ*_2_ norm in Eq. (3) is synonymous with the choice of variance as the measure of individual differences [15].

The variance in breeding value, *σ*_*A*_^2^, is called the *additive genetic variance*. The proportion of the total phenotypic variance, *σ*_*Y*_^2^, taken up by the additive genetic variance,

(5) $$h^{2}=\frac{\sigma_{A}^{2}}{\sigma_{Y}^{2}}\text{,}$$

is called the *narrow-sense heritability* of the phenotype under consideration. When writers refer to “missing heritability,” they mean the discrepancy between estimates of Eq. (5) from studies of pedigrees and the percentage of the variance ascribable to phenotype-associated SNPs identified with high confidence in GWAS. Below, we will describe new methods for estimating *h*^2^ and a means of identifying more of the SNPs contributing to this quantity.

In general, the weights in Eq. (2) are a difficult-to-compute function of the non-additive residuals, allele frequencies, and the correlation structure of polymorphic sites in the genome [14]. But it is of interest to examine the simplified case of a biallelic site that is uncorrelated—in *linkage equilibrium* (LE)—with all other causal sites and is itself in Hardy–Weinberg equilibrium. Let *p*_1_ and *p*_2_ denote the respective frequencies of $A_{1}$ and $A_{2}$. Suppose that we perform our hypothetical mutagenic experiment on a randomly sampled gamete carrying a gene of the $A_{1}$ allelic class. With probability *p*_1_ its partner gamete will also carry $A_{1}$, and with probability *p*_2_ its partner gamete will carry the alternative $A_{2}$. The expected effect of the gene substitution is thus

(6) $$\frac{p_{1}\Delta Y_{A_{1}\to A_{2}|\mathrm{other}\;\mathrm{gene}\;\mathrm{is}\;A_{1}}+p_{2}\Delta Y_{A_{1}\to A_{2}|\mathrm{other}\;\mathrm{gene}\;\mathrm{is}\;A_{2}}}{p_{1}+p_{2}}\text{,}$$

and it happens that in this case the weights (*p*_1_, *p*_2_) are precisely those leading to Fisher's average effect of gene substitution [16]. In reality it is likely that a causal site will be in linkage disequilibrium (LD) with other causal sites clustering near the same coding region. Distant causal sites may also be in very slight LD as a result of assortative mating or natural selection [14], [15], [17]. Nevertheless we think that the appealingly simple Eq. (6) will rarely give a poor approximation of the true average effect of gene substitution at a biallelic site.

## 3 The Linear Model of Quantitative Genetics

The concept of average effect is encapsulated in the linear model

(7) Y=Xα+R+E,

where **Y** is the vector of phenotypes, **X** is the genotype matrix, **R** is the vector of genetic residuals and **E** is the vector of non-genetic (“environmental”) residuals.

We have tacitly assumed the absence of any correlation between the non-genetic residuals and any column of **X**. Such confounding must be absent or remediable if we are to use empirical regression analysis to estimate the elements of *α*, as defined causally above. The inability to address analogous forms of confounding has been a bane to many fields of science limited to observational data [18]. A remarkable feature of GWAS, however, is that the correlation between the non-genetic residual and any given SNP is indeed often negligible [19]. We can point to a variety of checks supporting this claim, but perhaps the simplest and most convincing such check is the agreement between estimates of effects from samples of unrelated individuals and estimates from within families [5], [8], [20]. Recall that among the gametes produced by the same heterozygous parent, the allelic class of the transmitted allele is randomly selected and thus equivalent to treatment status in a randomized experiment [21], [22]. A positive result in a within-family study thus provides powerful evidence that a SNP is indeed linked and associated with a site where the average effect is nonzero.

A potential objection to the linear model of quantitative genetics, which features coefficients that are averages over a large number of contexts, is that it sacrifices too much of biological interest for dubious gain. Holders of such a position tend to emphasize the importance of the full genetic architecture as represented by Eq. (1), although as a concession to the problem of combinatorial explosion they often begin with simplifying strategies such as limiting the first-pass analysis to pairwise interactions [23], [24], [25].

An important preliminary point is that scans for linear average effects (more or less standard GWAS practice) will not necessarily preclude the detection of causal sites that interact nonlinearly with each other. In order for a site involved in an epistatic interaction to exhibit an average effect equaling zero, the various terms in Eq. (2) must mutually cancel, which is an extremely unlikely occurrence.

The detection of sites with nonzero average effects thus serves as an excellent starting point even if the investigator's ultimate goal is the characterization of epistasis. There is an important respect, however, in which epistasis (defined in this quantitative–genetic sense) is less biologically significant than average effects. It turns out that nonlinear interactions do not make substantial contributions to familial resemblance.

Fig. 1 demonstrates this point in the case of a single causal site. The dominance deviations—nonlinear deviations of the conditional phenotypic means of the three genotypes from their corresponding breeding values—do not enter the correlations between ancestors and descendants [15]. To explain this remarkable fact, we start with the observation that dominance deviations are equivalent to the residuals in the least-squares linear regression of the conditional means on gene count. The residuals in any linear regression have an expected value of zero; the values of the outcome variable will show no systematic tendency to lie either above or below the regression line. If Hardy–Weinberg equilibrium holds, we can write this fact as

(8) $$\sum_{i,j}p_{i}p_{j}\delta_{ij}=0\text{,}$$

where *δ*_*ij*_ is the dominance deviation of the genotype with alleles $A_{i}$ and $A_{j}$ with respective probabilities *p*_*i*_ and *p*_*j*_. Eq. (8) can be partitioned into terms that individually equal zero [26], [27]. That is,

(9) $$\sum_{j}p_{i}p_{j}\delta_{ij}=0\;for\;\mathrm{each}\;i\text{,}$$

which can also be put in the following way. In a subpopulation consisting of all individuals inheriting a particular allele (say $A_{1}$) from a given parent (say the father), the mean of the dominance deviations is zero—just as in the population as a whole. The geometry of Fig. 1 should make this plausible. Since adjacent dominance deviations have opposite signs, the frequency-weighted sum of dominance deviations after fixing one allele will intuitively tend to cancel and in fact does so exactly.

Let us say that $A_{1}$ is the allelic class of the gene that a parent transmits to its offspring. Under random mating the other gene at each individual's locus can be treated as drawn randomly from the entire population of genes. To simplify the notation, we now use *p* and 1 − *p* to denote the respective frequencies of $A_{1}$ and $A_{2}$. With probability (1 − *p*)^2^, parent and offspring have the same dominance deviation *δ*_11_. Similarly, with probability 2*p*(1 − *p*) they have different deviations (*δ*_11_ and *δ*_12_), and with probability *p*^2^ they share the heterozygous deviation (*δ*_12_). Observe that

(10) $$\begin{array}{l} Cov\left(\delta^{\left(\mathrm{parent}\right)},\delta^{\left(\mathrm{offspring}\right)}\right)={\left(1-p\right)}^{2}\delta_{11}^{2}+2p\left(1-p\right)\delta_{11}\delta_{12}+p^{2}\delta_{12}^{2}\\ \;=\delta_{11}\left[{\left(1-p\right)}^{2}\delta_{11}+\left(1-p\right)p\delta_{12}\right]+\delta_{12}\left[p\left(1-p\right)\delta_{11}+p^{2}\delta_{12}\right]\\ \;=\delta_{11}\cdot 0+\delta_{12}\cdot 0\\ \;=0\text{.} \end{array}$$

It follows that the correlations between the phenotypes of ancestors and descendants are exactly the same regardless of whether the conditional phenotypic means of the possible genotypes actually lie on the line determined by the average effect or deviate nonlinearly.

This absence of nonlinear contributions to ancestor–descendant correlations does not generalize to all other forms of residual (non-additive) genetic variance. In particular, when there are interactions among genes at different loci, these can alter the correlations between relatives. However, these epistatic variance components have coefficients in the expression for a given correlation that decrease geometrically with the order of the interaction, and thus the great bulk of the contribution to the resemblance between relatives (other than monozygotic twins) continues to be made by the additive genetic variance. And this brings us to a commonsensical observation: if individual differences were caused primarily by non-additive genetic differences, then relatives would not strongly resemble each other, but it is unquestionably true that in our world relatives *do* resemble each other. This simple fact points to the importance and size of *h*^2^, the proportion of the phenotypic variance due to variance in additive genetic value.

Given the undoubted importance of physical interactions between gene products in biological pathways, why do we not observe a more prominent role of epistasis in the genetic architectures of quantitative traits? One answer is that the typical allele frequencies at polymorphic sites may suppress the effects of the interactions that do occur. Once a new allele appears by mutation, the amount of time that it spends at each possible frequency *p* between zero and one before absorption at one of these two boundaries should be roughly proportional to 1/*p*[28], which means that we are much more likely now to observe the mutant when it is rare rather than common. This implies in turn that any genotype composed of many rare alleles must be much less common than its alternatives. One can appreciate the resulting tendency to linearize the genotype–phenotype mapping by inspecting Fig. 1. Suppose that the frequency of $A_{2}$ evolves to be close to zero rather than 0.6. Then the homozygous genotype $A_{2}A_{2}$ will be so rare as to be given virtually no weight in the least-squares regression determining the average effect, and the regression line will then have to fit essentially only two points. An almost perfectly additive genetic architecture will have evolved out of an intrinsically nonlinear arrangement of the three conditional means. Likewise, in the case of multiple sites, the frequency spectrum of mutant alleles ensures that the least-squares hyperplane does not have to fit as many points as we might naively think [29], [30]. Nonlinear architectures can be specially constructed to defeat this basic argument [24], but they require fine tuning [31].

Another answer is suggested by the striking concordance of GWAS findings across distinct populations. For instance, genetic effects from studies of East Asians are strongly correlated with estimates from studies of Europeans [32]. Because separately evolving populations differ in allele frequencies and LD patterns, the weights defining their respective average effects in Eq. (2) may be quite different. It seems to us that the simplest explanation for the agreement of the respective weighted averages despite the likely divergent weights is that the dependence on genomic background in Eq. (1) is often not very strong. This inference is explicable in light of a robust empirical regularity gleaned from GWAS: the individual effects of sites with common variants on a typical quantitative trait are quite small, often failing to account for even 1% of the phenotypic variance [2], [33], [34], [35]. The heritability of a typical quantitative trait is thus spread across thousands of genomic sites, each accounting for a very small portion of Var(*A*). A fair conclusion to draw from this trend is that variation at a typical causal site perturbs the relevant biological system by a small amount. The smallness of individual effects implies even smaller nonlinear deviations from strict additivity [36].

## 4 Estimation of Heritability Using Unrelated Individuals

Having established that the average effect is the biologically relevant quantity to estimate, we now address how such quantities are estimated. The most straightforward approach is to estimate the average effects in Eq. (7) directly by regressing the phenotypes of a population against their genotypes. However, in real applications the number of imputed or sequenced polymorphic sites *p* will typically exceed the number of individuals in the dataset *n*. In so-called *p* > *n* problems of this kind, the partial regression coefficients are not identifiable with ordinary least squares. In the next section, we show how the statistical theory of compressed sensing can be applied to directly estimate the individual average effects in the *p* > *n* regime. Here, we show how an important aggregate quantity—*h*^2^, the proportion of the phenotypic variance due to all genomic sites with nonzero average effects—can be estimated without knowledge of the individual sites contributing to this aggregate.

Classical methods of quantitative genetics estimate *h*^2^ by determining the extent to which the correlations between relatives increases with the degree of biological relatedness. Under some simplifying assumptions the correlation between relatives is given by

(11) $$\mathrm{Corr}\left(Y^{\left(\mathrm{relative}\;i\right)},Y^{\left(\mathrm{relative}\;i^{\prime }\right)}\right)=A_{i,i^{\prime }}h^{2}\text{,}$$

where $A_{i,i^{\prime }}$ is a coefficient that depends on the pedigree relationship. For example, the coefficient equals unity if the relatives are monozygotic twins, 1/2 if they are parent and offspring, 1/4 if they are uncle (aunt) and nephew (niece), and so on.

The use of Eq. (11) to estimate *h*^2^ from empirical correlations between relatives is often thought to be problematic because of the possibility that relatives resemble each other not only for genetic reasons but environmental ones [24]. This concern is probably overstated [37], but it is important to devise alternative estimators of *h*^2^ so as to minimize the possibility that the so-called missing heritability is attributable to biases of pedigree studies.

Classical methods based on the correlations between relatives have been substantially augmented by a novel technique that makes use of GWAS data from nominally unrelated individuals [38], [39]. This technique—often called *genomic-relatedness-matrix restricted maximum likelihood* (GREML) (we list URLs for all software tools at the end)—is perhaps the most important innovation in quantitative genetics to have been introduced in the last dozen years, and it has provided nearly definitive evidence for the view that undiscovered sites with common alleles account for a substantial portion of missing heritability.

For the moment we redefine the additive genetic variance, *σ*_*A*_^2^, to mean the variance that would be removed from the total phenotypic variance by multiple regression on all markers genotyped, sequenced, or imputed in a given study, as sample size goes to infinity. Because causal sites with a rare allele may not be present or represented by LD proxy in a given study, this additive genetic variance is less than the true additive genetic variance contributed by all polymorphic sites in the genome that we defined previously. Likewise, a site with a nonzero partial coefficient in the multiple regression now under consideration may not be a true causal site with a nonzero average effect but only an LD proxy for such a site. For convenience, however, we continue to use the terms “additive genetic variance,” “heritability,” “average effect” and their corresponding symbols in what follows.

We see from Eq. (7) that the total phenotypic variance can be written as

(12) $$\begin{array}{l} Var\left(Y\right)=\frac{1}{n}E\left(Y^{\prime }Y\right)\\ \;=\frac{1}{n}E\left(\alpha^{\prime }X^{\prime }X\alpha +e^{\prime }e\right)\\ \;=\sigma_{A}^{2}+\sigma_{E}^{2}\text{,} \end{array}$$

where **e** = **R** + **E** and the expectation is over random **e**. As before, the heritability is *h*^2^ = *σ*_*A*_^2^/(*σ*_*A*_^2^ + *σ*_*E*_^2^). If we assume that LE holds approximately, then **X**′**X** ≈ *n***I**_*p*_ and the additive genetic variance is approximately *α*′*α*. We can see that Eq. (12) holds because (1/*n*)*E*(**u**′**Z**′**Zu**) is the variance of chip-based breeding values and hence equal to *σ*_*A*_^2^.

The goal is to estimate *σ*_*A*_^2^ given **X** and **Y**. GREML treats Eq. (7) as the mixed-effects linear model

(13) $$\begin{array}{l} E\left(YY^{\prime }\right)=E\left(X\alpha \alpha^{\prime }X^{\prime }+ee^{\prime }\right)\\ \;≃A\sigma_{A,\mathrm{GREML}}^{2}+I_{n}\sigma_{E,\mathrm{GREML}}^{2} \end{array}$$

and estimates the parameters *σ*_*A*,GREML_^2^ and *σ*_*E*,GREML_^2^, where, in the notation of [38], **A** = (1/*p*)**XX**′ is the matrix of realized relatedness coefficients.

Eq. (13) is appealing because it assumes the same form as Eq. (11), except that the theoretical coefficient derived from the pedigree connecting biological relatives *i* and *i*′ is replaced by the chance genetic similarity (which is either slightly greater or slightly less than a mean of zero) between essentially unrelated individuals [40]. Because the slight genetic similarities between unrelated individuals in a homogeneous population are not likely to be correlated with environmental similarities, it becomes safer to make the assumption above that breeding values are uncorrelated with the total residuals.

Despite the surface similarity between Eqs. (11), (13), *h*^2^ and *h*_GREML_^2^ are not necessarily equal even under the same conditions that render Eq. (11) an unbiased estimator of *h*^2^[41]. The GREML Eq. (13) implicitly assumes that the outer product *αα*′ can be replaced by a diagonal matrix with all elements equal to the inner product *α*′*α*. As shown in [42] a sufficient condition for this approximation to be valid and as a result the equality of *h*^2^ and *h*_GREML_^2^ is that all sites are in LE. In practice, the two quantities will be very close if the causal sites are distributed randomly across the genome with respect to LD [42]. In other words, it must be the case that the extent of a site's LD with neighbors provides no information about its average effect (which may be zero). Since it is likely that causal variants tend to have lower minor allele frequencies (and hence are less well tagged by neighbors than a typical genotyped SNP) as a result of natural selection [33], [35], we will usually have *h*_GREML_^2^ < *h*^2^. A number of methods have been proposed to bring these two quantities into close agreement regardless of minor allele frequency and LD [43], [44], [45]. It appears that the most robust means of addressing this issue is to form several different relatedness matrices, stratifying the SNPs by LD, and then to estimate the additive genetic variance as the sum of the scalars weighting the LD-defined relatedness matrices in the natural extension of Eq. (13)[46].

The GREML method and variants have been used to estimate the heritabilities of several human traits and also the genetic correlations between them. The *genetic correlation* is simply the correlation between the breeding values with respect to two phenotypes. [47] gives the model for estimation of the genetic correlation between two traits and [48] for the entire genetic correlation matrix of arbitrarily many traits. The multivariate applications of the GREML method have led to some of its most interesting results. For instance, it turns out that the genetic correlation between schizophrenia and bipolar disorder approaches 0.70 [49].

One advantage of GREML-type methods for heritability estimation over classical pedigree-based methods is that the former can partition heritability among different regions of the genome. Partitioning by chromosome has shown that the heritability contributed by each chromosome is often strongly correlated with its length [8], [50], providing yet further evidence that the number of sites with nonzero average effects is typically very large. Partitioning by functional annotation has suggested that causal sites are disproportionately found in the vicinity of regions that are protein coding or DNase I hypersensitive [51]. Since the accuracy of the partitioning depends on the thoroughness of the imputation, these results should be taken as tentative. It is worth noting that both multivariate estimation and functional partitioning are more robust against LD than simple univariate estimation because of a tendency for biases to cancel from the numerators and denominators of the various estimands.

Very recently, a new method called *LD Score regression* has been introduced, and it can be put to some of the same uses as GREML [52], [53], [54]. When the chi-square statistics of the SNPs tested in a given GWAS are regressed against the “LD Scores” of the SNPs—the LD Score being a measure of the extent to which the focal SNP is in LD with its neighbors—the empirical result is an upwardly sloping straight line. This pattern is explicable in light of the fact that a SNP tagging more of its neighbors is thus more likely to tag one or more causal sites. Heuristically one might expect the value of the positive slope to provide an estimate of the trait's heritability, but the same GREML assumption regarding the absence of any relationship between average effect and LD must also hold for a valid estimate of *h*^2^ to be obtainable from LD Score regression. (Others conditions may also be necessary.) For instance, if high-LD genomic regions tend to be devoid of causal SNPs, then the slope of LD Score regression will be biased downward (and the intercept biased upward).

In fact, the first use of LD Score regression suggested by its developers is not the estimation of heritability but rather the control of confounding. This use follows from the interpretation of the intercept as the expected chi-square statistic of a SNP with an LD Score of zero. The lowest possible LD Score of a SNP is in fact one, which is obtained when a SNP is in perfect LE with all other SNPs. This essentially means that a hypothetical SNP with an LD Score of zero fails to tag the average effect of any SNP in the genome, including whatever average effect the SNP itself may have. Therefore, if the intercept of LD Score regression departs upward from unity (the theoretical expectation of the chi-square distribution with one degree of freedom), the departure must be due to confounding, poor quality control, sample overlap, or other artifacts. This simple and ingenious method of estimating the distribution of truly null SNPs should in most cases lead to a much better global inflation of the association statistics than the overly conservative genomic control [55].

We close this section with some practical recommendations. In as-yet unpublished work, we have found that LD Score regression can return different heritability estimates than GREML even when applied to the same data. Thus, when the purpose is to estimate the heritability of a phenotype, GREML is the tool of choice since it is unbiased or can be made to be nearly so. In contrast, when the purpose is functional partitioning of heritability, we strongly recommend LD Score regression over GREML because the former method scales much better computationally with the number of categories to which the heritability is allocated. LD Score regression can also estimate a genetic correlation from the association *Z*-statistics of two traits, and here it also offers many advantages over GREML: computational speed, input consisting of summary statistics rather than individual-level data, and absorption of confounding into the intercept. So far LD Score regression has produced estimates of genetic correlations very similar to those yielded by GREML [54], and in our unpublished work it has also produced estimates very similar to those of an intuitive in-house method that is based on the simple correlation between the two vectors of marginal regression coefficients. As is the case with GREML, functional partitioning and bivariate estimation with LD Score regression are more robust than simple heritability estimation because of a tendency for biases to cancel from numerator and denominator.

## 5 Finding Trait-associated Genetic Markers With Compressed Sensing

For the vast majority of phenotypes studied so far, the majority of the sites with nonzero average effects contributing to the heritability have not yet been identified. We now discuss a particular means by which progress toward this goal might be advanced.

A typical GWAS evaluates millions of polymorphic sites (*p*). The number of subjects (*n*) is increasing dramatically, but *p* > *n* will probably continue to hold for some time. As we stated earlier, the partial regression coefficients are not identifiable in this regime. Partly for this reason, GWAS investigators usually perform separate univariate regressions of their phenotype on each SNP and take forward the marginal coefficients obtained in this way. This approach is inherently unsatisfying, however, because the concepts of average effect and heritability rest on the partial coefficients. Therefore there is value in introducing some constraint (assumption) to deal with the ill-posed *p* > *n* problem in the GWAS setting.

The Bayesian approach known as *genomic selection* (GS) depends on a prior distribution quantifying the assumption that most of the SNPs in a given panel have no average effect. A major drawback of this approach is the heavy computational cost of sampling methods for estimating the parameters of a Bayesian model. Reference [56] applied an approach based on combinatorial geometry and random matrix theory called *compressed sensing* (CS) [57], [58], [59], which, in contrast to the Bayesian approach, requires little more than the computationally tractable minimization of the lasso objective function

(14) $${\left\|\hat{Y}-Y\right\|}_{\ell_{2}}^{2}+\lambda {\left\|\hat{\alpha }\right\|}_{\ell_{1}}\text{,}$$

where **Ŷ** is the estimated breeding value given by $X\hat{\alpha }$. The optimal choice of *λ* depends on the heritability contributed by the SNPs assayed in the study, which can be estimated with GREML. The minimum of Eq. (14) over $\hat{\alpha }$ can be found efficiently with the pathwise coordinate optimization (PCO) algorithm [60]. In the case of LE, PCO has the same computational complexity as the standard GWAS approach, *O*(*np*). LD increases the number of computations by either a constant or an amount that increases slowly with *p* (consistent with log *p*). A memory-efficient implementation of lasso employing PCO is available in the latest version of PLINK [61].

Suppose that the number of nonzero elements in the true *α* is equal to *s*. CS theory shows that under fairly general conditions, if *n* is sufficiently large compared to *s*—but, crucially, not necessarily larger than *p* and perhaps much smaller—then the lasso or other *ℓ*_1_-penalized schemes can select *all* polymorphic sites with nonzero coefficients in a multiple regression problem with high probability. (There is a major qualification, which we will explain shortly.) More specifically, if the sample size *n*′ < *n* is treated as a free parameter, then successive applications of the lasso to increasingly larger subsets of the data will result in a sharp transition from very poor selection to excellent selection. This transition can be observed in the behavior of the *P*-values returned by the standard univariate regressions of the phenotype on each of the SNPs selected by the lasso.

The CS approach makes no assumption about the distribution of the average effects. Instead it implicitly attempts to confine the estimate $\hat{\alpha }$ to an *s*-dimensional subspace. That is, if the true *α* in fact has *s* ≪ *p* nonzero elements, then these will be recovered by the lasso with high probability. There is evidence that, at least among sites where both alleles are common, *s* ≪ *p* for a wide range of traits [62], [63]. Since *n* is expected to exceed *s* by a large factor even while falling well short of *p*, the prospects of recovering more heritability are quite promising, especially in light of the current push to generate large and widely available datasets. Note that although there is a relationship between *ℓ*_1_-constrained solvers and the double Laplace prior that is debated in GS, CS theory is not based on this and holds for many different coefficient distributions and design matrices [57].

Finally, a given SNP is often strongly correlated—in tight LD—with several neighboring SNPs in the genome. This raises an obvious problem for the standard GWAS approach, since a causal SNP will lead many neighboring SNPs to exhibit nonzero univariate regression coefficients. The lasso does not in fact solve this problem. Although the lasso is statistically consistent under fairly general conditions, it may require a prohibitively large sample size to select only the causal sites in an LD block while setting the coefficients of all other sites to zero. Thus, in the presence of LD, “good recovery” means the selection of many sites that are false positives strictly speaking but nevertheless are in strong LD with one or more sites where the average effect is truly nonzero [56]. It is likely that no approach relying on statistical evidence alone can adequately address the problem of identifying the causal sites; external sources of biological evidence will be necessary. Particularly promising are empirical-Bayes approaches that use the trait-specific genome-wide relationship between GWAS signal and functional annotations (e.g., nonsynonymous status, tissue-specific DNase I hypersensitivity, chromatin modification, evolutionary conservation) to upweight the posterior probability of causality at certain sites [64], [65].

## 6 Summary and Outlook

In this review we have argued that the average effect of gene substitution—a weighted average of the phenotypic changes that would result from idealized mutagenic experiments—is the pivotal quantity to be estimated in GWAS. Although this averaging may conceal important nonlinear effects of genetic variation on the focal phenotype, the identification of sites with nonzero average effects is at least an important starting point. In any event new methods of heritability estimation based on DNA-level data confirm classical findings from the correlations between relatives that much phenotypic variation is attributable to the average effects of gene substitution across all causal sites. Pinning down all of this additive genetic variance to individual locations in the genome with high confidence continues to be a challenge, since the average effects are typically very small, but the theory of CS provides reason to believe that a transition to good recovery is attainable with a combination of *ℓ*_1_-penalization and large but reasonably realistic sample sizes.

Lurking not so far in the background behind all of these issues are the complications introduced by LD. Even if an oracle reveals to us the identity of a true causal site, that site's univariate regression coefficient may fail to equal its average effect of gene substitution as a result of LD. Perhaps a far more important concern is that LD prevents easy identification of causal sites responsible for GWAS signals in the first place. Furthermore, LD raises problems for GREML-type methods of heritability estimation that can probably stand further scrutiny. Notwithstanding these issues, however, the remarkable progress in quantitative genetics over the last decade leaves little doubt about the bountifulness of this research frontier.

**URLs**

GCTA-GREML, http://cnsgenomics.org/software/gcta;

LD Score regression, http://www.github.com/bulik/ldsc;

PLINK, https://www.cog-genomics.org/plink2.
